# Supplementary material for: Vegetation changes in temperate ombrotrophic peatlands over a 35 year period
Source: PLoS One. 2020 Feb 13;15(2):e0229146. doi: 10.1371/journal.pone.0229146 (PMC7018058; doi:10.1371/journal.pone.0229146)
Supplement: S1 Table — (DOCX) [file pone.0229146.s001.docx]

**S1 Table.**

| Plot | Latitude | Longitude | Plot | Latitude | Longitude |
| --- | --- | --- | --- | --- | --- |
| 1121 | 45.08296 | -73.17301 | 1202 | 46.27869 | -72.08002 |
| 1122 | 45.08430 | -73.16839 | 1203 | 46.60312 | -71.77081 |
| 1123 | 45.08726 | -73.17439 | 1204 | 46.60153 | -71.77515 |
| 1124 | 45.07751 | -73.17396 | 1207 | 46.59896 | -71.78168 |
| 1125 | 45.08618 | -73.17866 | 1208 | 46.59415 | -71.78432 |
| 1127 | 45.08082 | -73.17568 | 1210 | 46.41608 | -71.55428 |
| 1130 | 45.03220 | -73.25654 | 1211 | 46.40488 | -71.54038 |
| 1142 | 45.12582 | -74.21844 | 1212 | 46.39837 | -71.56024 |
| 1149 | 45.13709 | -74.28181 | 1215 | 46.37850 | -71.81285 |
| 1150 | 45.13722 | -74.21264 | 1216 | 46.79755 | -71.03869 |
| 1151 | 45.13825 | -74.21552 | 1217 | 46.79216 | -71.03751 |
| 1153 | 45.14140 | -74.28890 | 1218 | 46.78817 | -71.04513 |
| 1160 | 45.13049 | -74.22451 | 1219 | 46.78635 | -71.03828 |
| 1161 | 45.13264 | -74.22413 | 1220 | 46.78738 | -71.03359 |
| 1133 | 45.26037 | -73.02202 | 1221 | 46.77669 | -71.05054 |
| 1137 | 45.24338 | -73.04124 | 1222 | 46.77939 | -71.05225 |
| 1138 | 45.25734 | -73.02162 | 1223 | 46.78222 | -71.05779 |
| 1140 | 45.25154 | -73.02263 | 1224 | 46.77745 | -71.06037 |
| 1164 | 45.24064 | -73.03931 | 1225 | 46.77405 | -71.06607 |
| 1168 | 45.28931 | -73.03373 | 1226 | 46.78399 | -71.00117 |
| 1175 | 46.38824 | -71.53020 | 1228 | 46.77150 | -70.99397 |
| 1181 | 46.39041 | -71.53394 | 1229 | 46.76458 | -70.98478 |
| 1183 | 46.38997 | -71.54246 | 1230 | 46.78550 | -70.97253 |
| 1184 | 46.38434 | -71.53838 | 1231 | 46.78355 | -70.97846 |
| 1185 | 46.38234 | -71.62308 | 1232 | 46.79465 | -70.97210 |
| 1186 | 46.37951 | -71.62539 | 1233 | 46.79233 | -70.96725 |
| 1187 | 46.37869 | -71.63332 | 1234 | 46.79343 | -70.96308 |
| 1188 | 46.38742 | -71.56806 | 1240 | 46.87609 | -71.57076 |
| 1190 | 46.38924 | -71.55859 | 1241 | 46.87477 | -71.56645 |
| 1191 | 46.40045 | -71.55509 | 1242 | 46.87297 | -71.56580 |
| 1192 | 46.35793 | -71.03932 | 1243 | 46.87164 | -71.56837 |
| 1193 | 46.41478 | -71.54084 | 1244 | 46.87220 | -71.57431 |
| 1194 | 46.40987 | -71.53734 | 1245 | 46.87409 | -71.57858 |
| 1195 | 46.41140 | -71.53320 | 1246 | 46.87084 | -71.58240 |
| 1196 | 46.35492 | -71.03423 | 1247 | 46.86927 | -71.58064 |
| 1197 | 46.35387 | -72.02925 | 1248 | 46.86679 | -71.58663 |
| 1199 | 46.29221 | -72.05436 | 1249 | 46.77242 | -70.99904 |
| 1200 | 46.28856 | -72.04787 | 1250 | 46.75598 | -70.98322 |
