# Supplementary material for: Vegetation changes in temperate ombrotrophic peatlands over a 35 year period
Source: PLoS One. 2020 Feb 13;15(2):e0229146. doi: 10.1371/journal.pone.0229146 (PMC7018058; doi:10.1371/journal.pone.0229146)
Supplement: S2 Table — Taxa name, shade-tolerance (T = tolerant; M = mid-tolerant; I = intolerant), wetland indicator status (Obl = obligate; Fac = facultative; NW = non-wetland), frequency of occurrence for both years (number of plots) and in brackets mean abundance in the plots where the species is present are indicated. NA indicates inconsistent or unavailable information. (DOCX) [file pone.0229146.s002.docx]

**S2 Table.**

| Taxa name | Shade  tolerance | Wetland  status | Frequency  1982 | Frequency 2017 |
| --- | --- | --- | --- | --- |
| **TREES (8)** |  |  |  |  |
| *Abies balsamea* | T | NW | 1 (0.5) | 1 (37.5) |
| *Acer rubrum* | M | NW | 5 (1) | 25 (16.4) |
| *Betula papyrifera* | I | NW | 0 | 2 (0.5) |
| *Betula populifolia* | I | NW | 34 (8.5) | 27 (13.4) |
| *Larix laricina* | I | Fac | 58 (8.2) | 55 (7.9) |
| *Picea glauca* | M | NW | 0 | 1 (3) |
| *Picea mariana* | T | Fac | 57 (17.2) | 54 (17.6) |
| *Pinus strobus* | M | NW | 7 (1.2) | 7 (0.5) |
| **SHRUBS (28)** |  |  |  |  |
| *Alnus incana* subsp. *rugosa* | I | Fac | 8 (3.3) | 0 |
| *Alnus alnobetula* subsp. *crispa* | M | NW | 5 (9.7) | 0 |
| *Alnus* | NA | NA | 1 (3) | 0 |
| *Amelanchier bartramiana* | I | NW | 0 | 4 (1.1) |
| *Amelanchier* | NA | NA | 0 | 5 (0.5) |
| *Andromeda polifolia* | I | Obl | 25 (8.5) | 25 (4.5) |
| *Aronia melanocarpa* | M | NW | 0 | 33 (6.3) |
| *Betula pumila* | I | Obl | 22 (6.3) | 1 (0.5) |
| *Chamaedaphne calyculata* | I | Obl | 65 (27.4) | 61 (14.4) |
| *Frangula alnus* | M | NW | 0 | 3 (21.2) |
| *Gaylussacia baccata* | T | NW | 0 | 18 (11.9) |
| *Ilex mucronata* | M | Fac | 3 (2.2) | 26 (10.7) |
| *Kalmia angustifolia* | I | NW | 63 (10.7) | 63 (15.3) |
| *Kalmia polifolia* | I | Obl | 29 (7.6) | 41 (0.5) |
| *Prunus virginiana* | I | NW | 1 (3) | 1 (0.5) |
| *Rhododendron canadense* | T | Fac | 33 (11.7) | 42 (6.1) |
| *Rhododendron groenlandicum* | M | Obl | 60 (12.4) | 59 (3.4) |
| *Rubus allegheniensis* | I | NW | 0 | 4 (38.4) |
| *Rubus chamaemorus* | I | Fac | 2 (0.5) | 3 (1.3) |
| *Rubus hispidus* | I | Fac | 0 | 6 (2.9) |
| *Spiraea alba* var. *latifolia* | I | Fac | 1 (3) | 3 (12.8) |
| Undetermined ericaceous shrub | NA | NA | 1 (3) | 0 |
| *Vaccinium* cf. *angustifolium* | I | NW | 43 (12.3) | 62 (8) |
| *Vaccinium corymbosum* | T | Fac | 0 (0) | 18 (24) |
| *Vaccinium macrocarpon* | I | Obl | 0 (0) | 2 (0.5) |
| *Vaccinium oxycoccos* | I | Obl | 50 (3.8) | 51 (0.6) |
| *Vaccinium* | NA | NA | 4 (4.8) | 0 (0) |
| *Viburnum nudum* var. *cassinoides* | T | Fac | 1 (3) | 15 (0.8) |
| **HERBS AND FORBS (31)** |  |  |  |  |
| *Aralia nudicaulis* | T | NW | 0 (0) | 4 (1.1) |
| *Calla palustris* | M | Obl | 1 (3) | 0 |
| *Calopogon tuberosus* | NA | Obl | 0 | 4 (0.5) |
| *Carex* | NA | NA | 52 (18.1) | 52 (5.7) |
| *Clintonia borealis* | M | NW | 0 | 1 (15) |
| *Coptis trifolia* | T | Fac | 0 | 11 (2.3) |
| *Cypripedium acaule* | T | NW | 5 (1) | 42 (0.5) |
| *Drosera intermedia* | I | Obl | 1 (3) | 0 |
| *Drosera rotundifolia* | I | Obl | 10 (1.8) | 16 (0.8) |
| *Equisetum palustre* | I | Fac | 2 (1.8) | 0 |
| *Eriophorum angustifolium* | I | Obl | 0 | 3 (0.5) |
| *Eriophorum vaginatum* subsp. *spissum* | I | Obl | 62 (17.4) | 43 (2.8) |
| *Eriophorum virginicum* | I | Obl | 0 | 17 (1.8) |
| *Gaultheria hispidula* | T | Fac | 1 (3) | 25 (1.8) |
| *Impatiens capensis* | M | Fac | 0 | 5 (0.5) |
| *Lycopodium annotinum* | T | NW | 0 | 1 (15) |
| *Lysimachia borealis* | T | NW | 0 | 9 (1.3) |
| *Maianthemum canadense* | T | NW | 0 | 9 (3.5) |
| *Maianthemum trifolium* | I | Fac | 21 (5.9) | 19 (1.7) |
| *Medeola virginiana* | T | NW | 0 | 2 (0.5) |
| *Monotropa uniflora* | T | NW | 0 | 3 (0.5) |
| *Nuphar variegata* | M | Obl | 0 | 5 (1) |
| *Osmundastrum cinnamomeum* | T | Fac | 0 | 5 (8.4) |
| *Platanthera blephariglottis* var. *blephariglottis* | NA | Obl | 0 | 14 (0.5) |
| *Poaceae* | NA | NA | 2 (7.8) | 0 |
| *Rhynchospora alba* | I | Obl | 0 | 2 (1.8) |
| *Rubus pubescens* | T | Fac | 0 | 4 (0.5) |
| *Sarracenia purpurea* | I | Obl | 39 (1.9) | 35 (0.5) |
| *Scheuchzeria palustris* | I | Obl | 0 | 2 (7.8) |
| *Utricularia cornuta* | I | Obl | 1 (37.5) | 0 |
| *Viola* | NA | NA | 0 | 3 (0.5) |
