# Supplementary material for: Vegetation changes in temperate ombrotrophic peatlands over a 35 year period
Source: PLoS One. 2020 Feb 13;15(2):e0229146. doi: 10.1371/journal.pone.0229146 (PMC7018058; doi:10.1371/journal.pone.0229146)
Supplement: S3 Table — (DOCX) [file pone.0229146.s003.docx]

**S3 Table.**

|  | 1982 |  |  | 2017 |  |  | 1982 | |  | 2017 | |
| --- | --- | --- | --- | --- | --- | --- | --- | --- | --- | --- | --- |
| Plot | Tree | Shrub |  | Tree | Shrub | Plot | Tree | Shrub |  | Tree | Shrub |
| 1121 | 15 | 87.5 |  | 0 | 87.5 | 1202 | 0 | 3 |  | 0 | 62.5 |
| 1122 | 3 | 37.5 |  | 62.5 | 62.5 | 1203 | 37.5 | 37.5 |  | 37.5 | 3 |
| 1123 | 37.5 | 62.5 |  | 37.5 | 37.5 | 1204 | 15 | 37.5 |  | 3 | 15 |
| 1124 | 15 | 62.5 |  | 15 | 62.5 | 1207 | 3 | 0.5 |  | 37.5 | 62.5 |
| 1125 | 0 | 15 |  | 62.5 | 37.5 | 1208 | 15 | 3 |  | 62.5 | 62.5 |
| 1127 | 15 | 62.5 |  | 15 | 87.5 | 1210 | 15 | 62.5 |  | 15 | 37.5 |
| 1130 | 37.5 | 62.5 |  | 62.5 | 62.5 | 1211 | 0 | 3 |  | 0.5 | 62.5 |
| 1142 | 0 | 3 |  | 0.5 | 87.5 | 1212 | 15 | 15 |  | 15 | 87.5 |
| 1149 | 0 | 62.5 |  | 0 | 87.5 | 1215 | 15 | 37.5 |  | 0.5 | 37.5 |
| 1150 | 0 | 0.5 |  | 0 | 87.5 | 1216 | 0 | 0.5 |  | 0.5 | 37.5 |
| 1151 | 0 | 37.5 |  | 0 | 87.5 | 1217 | 15 | 37.5 |  | 0.5 | 87.5 |
| 1153 | 0 | 3 |  | 0 | 87.5 | 1218 | 15 | 15 |  | 0 | 87.5 |
| 1160 | 0 | 15 |  | 0 | 87.5 | 1219 | 3 | 62.5 |  | 3 | 62.5 |
| 1161 | 0.5 | 0.5 |  | 0 | 87.5 | 1220 | 3 | 62.5 |  | 3 | 87.5 |
| 1133 | 15 | 15 |  | 37.5 | 87.5 | 1221 | 3 | 15 |  | 37.5 | 87.5 |
| 1137 | 62.5 | 62.5 |  | 62.5 | 87.5 | 1222 | 0 | 15 |  | 0.5 | 87.5 |
| 1138 | 15 | 15 |  | 3 | 62.5 | 1223 | 3 | 15 |  | 0.5 | 62.5 |
| 1140 | 15 | 15 |  | 3 | 87.5 | 1224 | 3 | 15 |  | 3 | 62.5 |
| 1164 | 37.5 | 37.5 |  | 62.5 | 62.5 | 1225 | 3 | 3 |  | 3 | 87.5 |
| 1168 | 0 | 0 |  | 62.5 | 62.5 | 1226 | 37.5 | 37.5 |  | 37.5 | 87.5 |
| 1175 | 3 | 15 |  | 3 | 62.5 | 1228 | 15 | 15 |  | 0.5 | 37.5 |
| 1181 | 3 | 15 |  | 37.5 | 37.5 | 1229 | 0.5 | 37.5 |  | 37.5 | 62.5 |
| 1183 | 15 | 37.5 |  | 0.5 | 62.5 | 1230 | 3 | 15 |  | 0.5 | 62.5 |
| 1184 | 3 | 3 |  | 15 | 62.5 | 1231 | 0 | 3 |  | 3 | 87.5 |
| 1185 | 3 | 0 |  | 15 | 87.5 | 1232 | 3 | 3 |  | 15 | 62.5 |
| 1186 | 37.5 | 62.5 |  | 37.5 | 62.5 | 1233 | 3 | 15 |  | 15 | 62.5 |
| 1187 | 3 | 37.5 |  | 15 | 87.5 | 1234 | 3 | 62.5 |  | 15 | 87.5 |
| 1188 | 15 | 0.5 |  | 15 | 62.5 | 1240 | 3 | 15 |  | 0.5 | 37.5 |
| 1190 | 15 | 15 |  | 3 | 62.5 | 1241 | 3 | 15 |  | 0.5 | 37.5 |
| 1191 | 15 | 37.5 |  | 15 | 62.5 | 1242 | 3 | 15 |  | 0.5 | 37.5 |
| 1192 | 3 | 15 |  | 3 | 62.5 | 1243 | 3 | 15 |  | 3 | 62.5 |
| 1193 | 3 | 3 |  | 0.5 | 37.5 | 1244 | 15 | 37.5 |  | 37.5 | 15 |
| 1194 | 15 | 37.5 |  | 37.5 | 62.5 | 1245 | 37.5 | 37.5 |  | 3 | 37.5 |
| 1195 | 3 | 15 |  | 3 | 37.5 | 1246 | 37.5 | 62.5 |  | 3 | 37.5 |
| 1196 | 3 | 15 |  | 3 | 37.5 | 1247 | 15 | 15 |  | 0 | 62.5 |
| 1197 | 3 | 15 |  | 15 | 87.5 | 1248 | 0 | 3 |  | 0.5 | 37.5 |
| 1199 | 37.5 | 62.5 |  | 15 | 62.5 | 1249 | 0 | 3 |  | 3 | 15 |
| 1200 | 3 | 3 |  | 37.5 | 62.5 | 1250 | 0 | 3 |  | 0.5 | 15 |
